# Sustainable biosynthesized bimetallic ZnO@SeO nanoparticles from pomegranate peel extracts: antibacterial, antifungal and anticancer activities

**DOI:** 10.1039/d3ra03260d

**Published:** 2023-07-28

**Authors:** Amr H. Hashem, Abdulaziz A. Al-Askar, Mohammad Reza Saeb, Kamel A. Abd-Elsalam, Ahmad S. El-Hawary, Mohamed S. Hasanin

**Affiliations:** a Botany and Microbiology Department, Faculty of Science, Al-Azhar University Cairo 11884 Egypt amr.hosny86@azhar.edu.eg; b Department of Botany and Microbiology, Faculty of Science, King Saud University P.O. Box 2455 Riyadh 11451 Saudi Arabia; c Department of Polymer Technology, Faculty of Chemistry, Gdańsk University of Technology Narutowicza 11/12 Gdańsk Poland; d Plant Pathology Research Institute, Agricultural Research Center Giza 12619 Egypt; e Cellulose & Paper Department, National Research Centre El-Buhouth St. Dokki 12622 Egypt sido_sci@yahoo.com

## Abstract

Sustainable bimetallic nanoparticles (NPs) have attracted particular attention in the past decade. However, the efficiency and environmental concerns are associated with their synthesis and properties optimization. We report herein biosynthesis of bimetallic ZnO@SeO NPs based on green and ecofriendly methods using pomegranate peel extract (PPE). Pyrochemical ultraviolet-visible (UV-vis), Fourier-transform infrared (FTIR) and X-ray diffraction (XRD) spectroscopy as well as TEM and EDX supported successful synthesis. Antibacterial, antifungal, and cytotoxic activities were indicative of biological worth of sustainable bimetallic ZnO@SeO NPs, exhibiting antibacterial activity compared to monometallic ZnO and SeO NPs. The values of Minimum Inhibitory Concentration (MIC) of bimetallic ZnO@SeO NPs toward *E. coli*, *P. aeruginosa*, *B. subtilis* and *S. aureus* were 3.9, 15.62, 3.9 and 7.81 μg ml^−1^, respectively. Likewise, a promising antifungal activity against *Candida albicans*, *Aspergillus flavus*, *A. niger* and *A. fumigatus* was achieved (MICs: 31.25, 1.95, 15.62 and 15.62 μg ml^−1^, respectively). The cytotoxicity results suggest that bimetallic ZnO@SeO NPs are non-toxic and biomedically safe, evidenced by *in vitro* anticancer activity against human liver carcinoma (Hep-G2) cell line (with a half-maximal inhibitory concentration (IC50) > 71 μg ml^−1^). The bimetallic ZnO@SeO NPs successfully biosynthesized using PPE showed a high potential for biomedical engineering.

## Abstract 2

Sustainable bimetallic nanoparticles (NPs) have attracted particular attention in the past decade.

## Graphical abstract

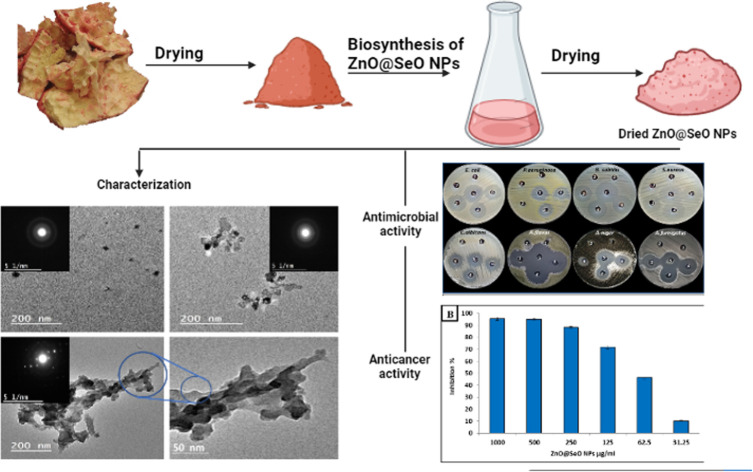

## 1. Introduction

Microbe-caused infections have emerged as a major cause of illness and mortality across the globe.^1^ Typically, microbes become resistant to antibiotics by producing enzymes that can modify, passivate, or worsen the antibiotic effects. Drug resistance can also arise as a consequence of bacterial alterations and modifications to efflux routes that hinder drug passage.^2^ Antibiotic-resistant bacterial illnesses have resulted in more than 1.27 million deaths in 2019.^3^ By 2050, it is predicted that more than 10 million people will die annually from diseases that are resistant to treatment in the case the current trends continue.^4^ In addition, among patients with compromised immune systems, fungal infections have considerably increased during the past ten years.^5^ Pathogenic fungi have infected more than 1.2 billion people worldwide, resulting in at least 1.7 million fatalities annually.^6^ There are currently around 750 000, 300 000, and 10 000 cases per year of invasive candidiasis, aspergillosis, and mucormycosis (also known as black fungus), respectively.^7^ The widespread use of antifungal medications results in the development of fungi resistant to most marketed antifungal medications.^8^ To this extent, the design of novel and highly efficient antifungal agents seems essential.

Nanoparticles (NPs) are commonly employed as an alternative to antibiotics to target microorganisms.^9^ Nanomaterials and nanostructures exhibit broad-spectrum antibacterial properties.^10–14^ Researchers have discovered that bimetallic NPs made of two distinct metals combine their antimicrobial capabilities compared to their monometallic counterparts.^15^ Due to the synergistic interaction between resulting from both monometallic parts, bimetallic NPs exhibit enhanced antimicrobial and anticancer efficacy.^16^ Depending on the synthesis pathway, microstructure, catalytic, and optical characteristics, bimetallic NPs can combat cancer. On the other hand, animal tests are indicative of the dependency of therapeutic effects of bimetallic NPs on the volume of tumors as well as the weight of mice.^17^ Thus, further investigations seem essential to explore all aspects of their therapeutic features. There is adequate evidence that the bimetallic NPs reveal antimicrobial synergetic effect, along with complementary cytocompatibility.^18–20^ TiO_2_/Au and ZnO/Ag are examples of bimetallic NPs with promising anticancer activity and low cytotoxicity.^21,22^ Another example is the plan-derived Cu/Zn bimetallic NPs which showed synergistic cytotoxicity effect when combined with anticancer doxorubicin against MCF-7 cancer.^23^

Sustainability concerns nowadays are centered on decision making loops in synthesis and biomedical engineering of NPs,^24,25^ it seems necessary to mobilize all agricultural practices in order to improve global production of fruits and vegetables in line with the global concerns about population growth. Additionally, varieties of fruits, such as banana, watermelon, papaya, mango, and pineapple, are widely regarded for their flavor and nutritional worth, but more than 40% of the mass of the fruit, including the peel, pulp, and seeds, is inedible.^26^ Fruit peel wastes are used for green biosynthesis of selenium NPs as orange peel,^27^ prickly pear peel,^28^ and pomegranate peel.^29^ In the strict sense of the word, the biosynthesis of NPs using plant extract is a promising strategy for may be restricted benefits including environmental and cost effects.^30^ Therefore, in the present work the crud pomegranate peel extract (PPE) was used to synthesize bimetallic ZnO@SeO NPs, which were compared for antibacterial, antifungal and anticancer activities with individual ZnO and SeO NPs. The comparative study included physiochemical and topographical studies as well as biological activity evaluation.

## 2. Materials and methods

### 2.1. Materials

Analytical grade chemicals such as zinc acetate, and selenium oxide were purchased from Sigma Aldrich, Germany. Microbiological media utilized in the following targeted tests were purchased from Oxoid, UK. Moreover, PPE was collected from juice factory in Obour city, Egypt.

### 2.2. Preparation of PPE and biosynthesis of bimetallic ZnO@SeO NPs, ZnO NPs and SeO NPs

PPE was prepared according to method used by.^31^ PPE was used for biosynthesis of bimetallic ZnO@SeO NPs, as well as ZnO and SeO NPs as the reference single metallic NPs through the following green method. In details, 2.0 mM of zinc acetate were mixed with 2.0 mM of selenium oxide and completely dissolved in 100 ml of the created PPE for biosynthesis of bimetallic ZnO@SeO NPs. Also, zinc acetate and selenium oxide were used separately for biosynthesis of ZnO NPs and SeO NPs, respectively. To synthesize bimetallic ZnO@SeO NPs, ZnO NPs, and SeO NPs, the reaction conditions must be pH 8.0, incubation temperature 30 °C, and reaction period 24 h under agitation (250 rpm) in shaking incubator.^32^ After incubation, the bimetallic ZnO@SeO NPs, ZnO NPs, and SeO NPs bioformed as dim brown. Finally, biosynthesized bimetallic ZnO@SeO NPs, ZnO NPs, and SeO NPs were cleared by centrifugation at 7500 rpm for 15 min and washed five times with distilled water to remove weakly bound peel biomolecules.

### 2.3. Characterizations

The comparative evaluation of the biosynthesized NPs was performed in terms of physiochemical analyses, including UV-vis spectroscopy (V-630 UV-vis spectrophotometer, Jasco, Japan) in the range of 1000–200 nm as well as the optical band gap of ZnO, SeO and ZnO@SeO NPs. The optical band gap was calculated using the following equation^22^ according to Tauc plot:(*αhν*)^*n*^ = *B*(*hν* − *E*_g_)where *hν* is the photo energy, *B* is a constant correlated to the specific material, *α* is the coefficient of absorption, and *n* takes the value of 2 and ½ for a direct and indirect transition, respectively.

FTIR spectroscopy (Nicolet Impact-400 FT-IR spectrophotometer) in the range of 4000–400 cm^−1^, and XRD that was investigated using a Diano X-ray diffractometer (Philips). Otherwise, the topographical study included field emission SEM Model Quanta 250 FEG and TEM Model JEM2010, Japan.

### 2.4. Antibacterial and antifungal activities

#### 2.4.1. Microbial strains and growth conditions

Antibacterial activity of ZnO, SeO, and ZnO@SeO NPs and other precursors was assessed against Gram-negative bacteria (*Escherichia coli* ATCC 25922 & *Pseudomonas aeruginosa* ATCC 27853), Gram-positive bacteria (*Staphylococcus aureus* ATCC 25923 & *Bacillus subtilis* ATCC 6051), Likewise, antifungal activity was evaluated against unicellular (*Candida albicans* ATCC 90028) and multicellular fungi (*Aspergillus niger* RCMB 02724, *A. flavus* RCMB 02782 and *A. fumigatus* RCMB 02568).

#### 2.4.2. Agar well diffusion method

The test of diffusion in agar was performed in accordance with document M51-A2 of the Clinical Laboratory Standard Institute^33^ with minor adaptations. The selected bacterial strains were grown on nutrient agar media for 24 h at a 37 °C. Bacterial suspensions with a concentration of 1.5 × 10^6^ colony forming units per milliliter (CFU ml^−1^) were individually prepared. These suspensions were then introduced into Muller Hinton agar media and carefully poured into sterilized Petri plates under aseptic techniques. A total volume of 100 μl of bimetallic ZnO@SeO, ZnO, and SeO NPs, as well as other initial precursors, along with a standard antibiotic (Amoxicillin/clavulanate) at a concentration of 1000 μg ml^−1^, was introduced into agar wells. Subsequently, the plates were refrigerated for 2 h, after which they were incubated at a temperature of 37 °C 24 h. On the other hand, fungal strains were grown on malt extract agar (MEA) plates and subjected to incubation at 30 °C for 3–5 days. The fungal suspension was prepared using a sterilized phosphate buffer solution (PBS) with a pH of 7.0. Subsequently, the inoculums were adjusted to a concentration of 10^7^ spores per milliliter after being counted in a cell counter chamber. A volume of 1 milliliter was evenly distributed across agar MEA plates. A sterile cork-borer with a diameter of 8 mm was used to create wells in the inoculated MEA plates. Subsequently, 100 μl of bimetallic ZnO@SeO NPs, ZnO NPs, SeO NPs, other initial precursors, and a standard antifungal agent (fluconazole) at a concentration of 1000 μg ml^−1^ were introduced into the wells. All MEA plates were incubated at 30 °C for 72 h. Finally, after ending the incubation period for both bacteria and fungi, the inhibition zone diameter for each treatment was measured.

#### 2.4.3. Minimum Inhibitory Concentration (MIC)

Different concentrations of ZnO@SeO, ZnO, and SeO NPs were prepared, ranging from 1000 to 1.95 μg ml^−1^, and then tested independently to determine MIC against particular bacterial and fungal species.^34,35^

#### 2.4.4. *In vitro* cytotoxicity and antitumor activity

The normal Vero cell line, and cancerous cell lines Hep-G2 (liver cancer) which collected from American type culture collection (ATCC) were used to check the cytotoxicity of ZnO@SeO NPs using MTT protocol^36^ with minor modification. A 96-well tissue culture plate was inoculated with a cell density of 1 × 10^5^ cells per milliliter, with 100 microliters per well. The plate was then incubated at a temperature of 37 °C for 24 h, allowing for the formation of a fully developed monolayer sheet. The growth medium was carefully poured out from the 96-well microtiter plates subsequent to the establishment of a confluent sheet of cells. The cell monolayer was then subjected to two rounds of washing using wash media. Serial dilutions of the tested sample were prepared in RPMI medium supplemented with 2% serum, which serves as the maintenance medium. Subsequently, a volume of 100 μl from each dilution was meticulously dispensed into distinct wells, while reserving three wells as control, which solely received the maintenance medium. The plate was then subjected to incubation at a temperature of 37 °C, followed by thorough examination. The cellular specimens were subjected to thorough examination in order to identify any observable manifestations of toxicity, such as the presence of partial or complete monolayer disruption, cellular rounding, reduction in size, or the occurrence of cellular granulation. A solution of MTT (5 mg ml^−1^) in PBS, provided by BIO BASIC CANADA INC, was prepared. Subsequently, 20 μl of the MTT solution were added to each well and agitated at a speed of 150 rpm for 5 min. This was followed by an incubation period at a temperature of 37 °C, in an atmosphere containing 5% CO_2_, for a total of 4 h. The purpose of this incubation was to facilitate the metabolic processing of MTT. The measurement of optical density was conducted at a wavelength of 560 nm. Plotting *x*–*y* and fitting the data with a straight line (linear regression) yields the simplest way to determine the IC50. The cell quantity and the percentage of viable cell were totaled by the following formula:
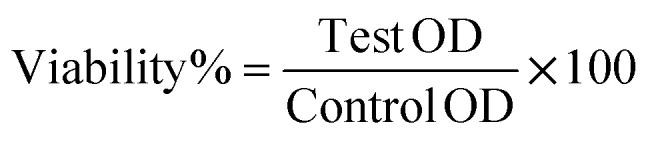
Inhibition% = 100 − viability %

### 2.5. Statistical analysis

The means of three replicates and standard errors were calculated for all obtained results, and the data were subjected to analysis of variance means using Minitab 17 software.

## 3. Results and discussion

### 3.1. Biosynthesis and characterization of bimetallic ZnO@SeO NPs, ZnO NPs and SeO NPs

PPE has been utilized to biosynthesize several metal NPs.^37,38^ Pomegranate peel contains significant levels of phenolic chemicals, including elements necessary for the formation of metal NPs, such as hydrolysable tannins, flavonoids.^39^ The current study employed PPE to synthesize bimetallic ZnO@SeO NPs, ZnO NPs, and SeO NPs. Then, faint red, white and red colors were observed which indicates biosynthesis bimetallic ZnO@SeO NPs, ZnO NPs, and SeO NPs, where PPE was acting as a reducing or capping agent. Plant extract bioactive phytochemicals act as a capping agent, inhibiting nanoparticle aggregation and modifying their biological activity.^40,41^

**Fig. 1:**
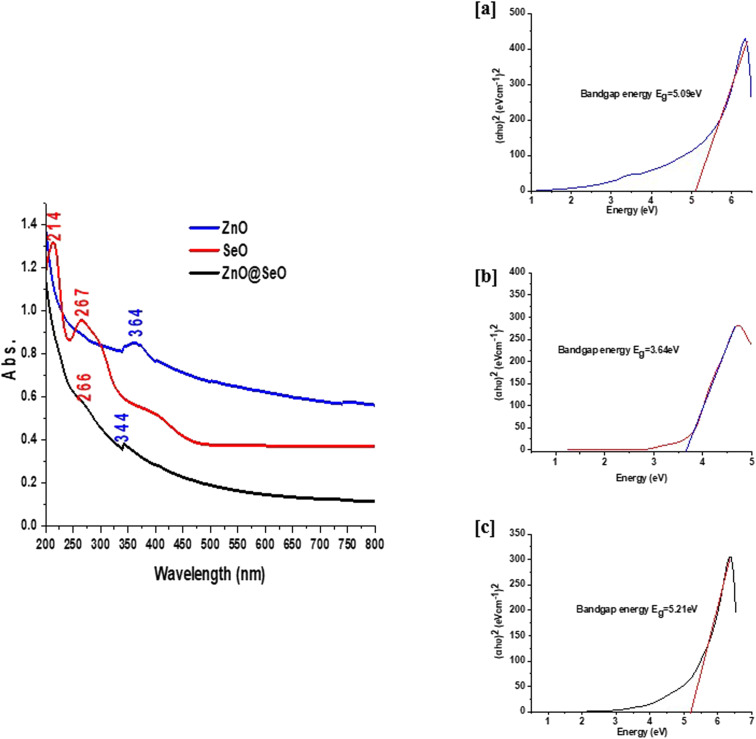
UV-visible spectra of bimetallic NPs and individual NPs (left) and the Tauc plot to find the band gap of ZnO NPs (a), SeO NPs (b) and (ZnO@SeO).

The characterizations of the biosynthesized NPs include individual and bimetallic NPs were carried out using UV-visible as shown in Fig. 1. The ZnO NPs spectrum observed a broad band at 364 nm that is characteristic of the absorbance of ZnO NPs in the UV range.^42,43^ Otherwise, the SeO NPs spectrum observed two characteristic bands at 214 and 267 nm as sharp bands.^44,45^ In this context, the bimetallic spectrum was detected of the broadness of ZnON Ps band and shifting to low frequency and assigned at 344 nm. Additionally, the SeO NPs appeared at 266 nm as small broadband and the other band at 214 nm declined as well. Indeed, the spectrum of ZnO@SeO illustrated a combination between both metal oxides and this reflected new features that popped up from the origin. In addition to, the band gap energy calculation *via* Tauc model (Fig. 1a–c) of biosynthesis NPs that individual and bimetallic were clarified a significant difference. The band gap of ZnO NPs (Fig. 1a) was 5.09 eV that referred to nanostructure of ZnO according to Wang *et al.*, 2011.^46^ Moreover, the SeO NPs band gap was presented in Fig. 1b was recorded as 3.64 eV that in a nice agreement with Khusayfan *et al.*, 2022.^47^ Otherwise, the band gap of ZnO@SeO bimetallic was increased to 5.21 eV in comparison with both individual nanoparticle values that could be due to the relationship between the bandgap of nanocrystals and effective mass charge carriers.^20^ With other words, the binding of the NPs could be attracted *via* a electrostatic force that tying the electrons and increase of the band gap value as well.

**Fig. 2:**
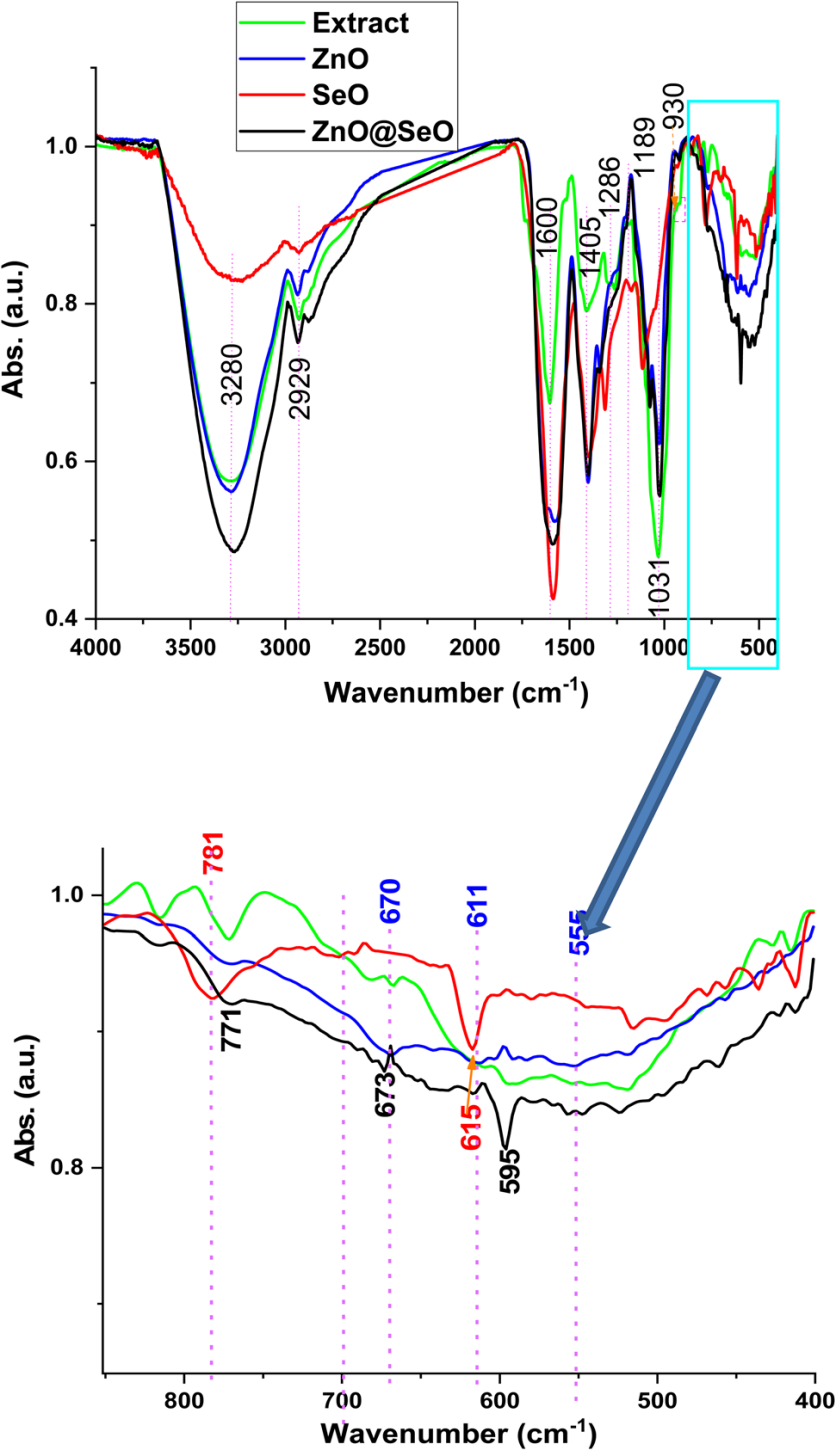
FTIR spectra of bimetallic NPs and individual NPs.

The FTIR spectroscopy in Fig. 2 was shown the comparative studied between PPE and biosynthesis individual and bimetallic NPs. The PPE spectrum was shown functionalized groups bands at 3280, 2929, 1600, 1405, 1286 and 1031 cm^−1^ are attributed to stretching vibrations of hydroxyl groups overlapped with primary amines, CH stretching vibrations overlapped with secondary amines, bending vibrations of amines, CH and CH_2_ aliphatic bending group, C–N stretching vibration of aromatic rings and C–O–C polysaccharide, respectively. Additionally, the band at 1189 cm^−1^ corresponds to phosphate groups of remaining nucleic acids, polyphosphates and phospholipids and band at 930 cm^−1^ is related to C=C, C=N, C–H in ring structure.^48–50^ The mentioned above function groups are consumed as a capping and stabilizer for the synthesis of NPs. On the other side, the biosynthesized NPs FTIR spectra observed significant and obvious changes after formulation. These changes went out as the same approximately for the changes in the extract function groups in all prepared NPs. Whereas the fingerprint FTIR region affirmed different changes according to metal oxide type. The ZnO NPs spectrum shown 670, 611 and 555 cm^−1^ were attributed to Zn–O stretching and deformation, respectively.^51,52^ In addition, the SeO NPs assigned at 781 and 615 cm^−1^ were related to Se–O.^53,54^ Herein, the bimetallic NPs 771, 673 and 595 cm^−1^ were attributed to the interaction of the bimetallic NPs. Indeed, these bands were due to the individual groups of both metal oxide with overlapping assigned obviously.

**Fig. 3:**
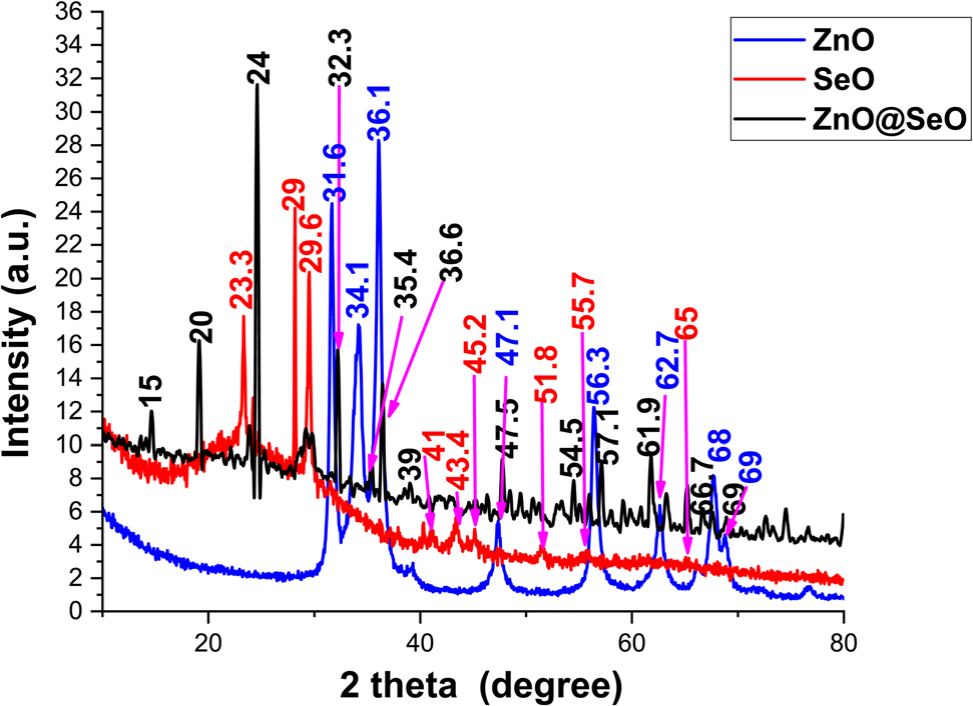
XRD pattern of bimetallic and individual NPs.

Fig. 3 shows the crystallographic pattern of biosynthesis NPs. ZnO NPs pattern was shown a diffraction peak at 31.6, 34.1, 36.1, 47.1, 56.3, 62.7, 68 and 69° that related to hexagonal wurtzite phase of ZnO (JCPDS card 36-1451).^55,56^ In addition, SeO NPs 23.3, 29, 29.6, 41, 43.4, 45.2, 51.8, 55.7 and 65° hexagonal structure were successfully formed, and the lattice constants were *a* = 4.36 Å and *c* = 4.95 Å as per (JCPDS card No. 06-362) standard.^44,57^ However, the bimetallic crystallographic pattern was assigned peaks at 15, 20, 24, 32.3, 35.4, 36.6, 39, 47.5, 54.6, 56, 57.1, 61.9, 63.4, 65.1, 66.7 and 69° were attributed to the interaction of ZnO NPs and SeO NPs. Moreover, the ZnO NPs and SeO NPs as well as diffraction were observed in a bimetallic pattern. Meanwhile, new peaks were observed in a bimetallic pattern at 15, 20, 33, 35, 39, 54.6, 57.1, 61.9, 63.4, 66.7 and 69° these peaks were related to intergradation of both ZnO NPs and SeO NPs. Moreover, the characteristics peaks of ZnO NPs was assigned obviously in bimetallic pattern at 36.6, 47.5, 56, 61.9 and 69° with some slightly division in comparison with the individual nanoparticle patten. Likewise, SeO NPs peaks were presented in bimetallic at 56° that overlapped between ZnO NPs and SeO NPs as well as 65.1°. Consequently, the XRD pattern affirmed the attraction between both metal oxide nanoparticle to formulated the bimetallic ZnO@SeO.

### 3.2. Morphological analysis

**Fig. 4:**
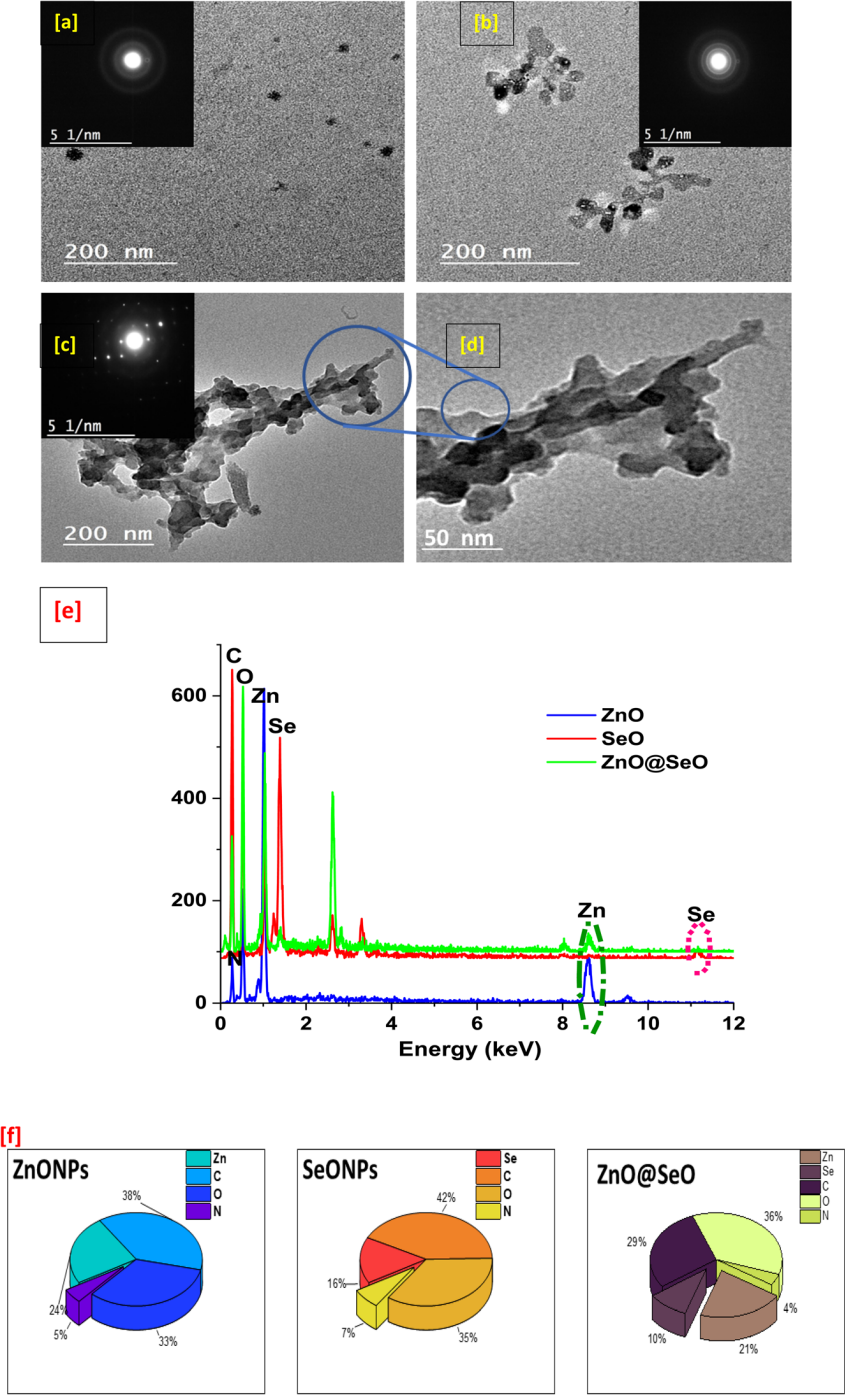
TEM of ZnO NPs (a), SeO NPs (b) and bimetallic (c and d) as well as EDX charts (e) and EDX element percentage (f).

The morphological study of biosynthesis NPs was observed using TEM, as shown in Fig. 4. The ZnO NPs TEM image (Fig. 4a) revealed a spherical particle with a size of around 20 nm; this shape could be hexagonal as extracted from the XRD pattern. Additionally, the SeO NPs image (Fig. 4b) showed a clearer particle with a nonregular spherical shape that was close to a hexagonal shape with a particle size of about 33 nm. These observations are in nice agreement with crystallographic study. On the other hand, the bimetallic NPs (Fig. 4c and d) were observed as a cluster aggregated together with a good distribution in size and an unformal shape related to SeO NPs. Moreover, the bimetallic particle shown as a melted particle in a low magnification image (Fig. 4c) and the high magnification image (Fig. 4d) emphasized the particle shape with a size of around 40 nm.

Furthermore, the tested samples shown carbon, nitrogen, and oxygen in EDX chart (Fig. 4e) as essential elements components. However, the atoms Zn and Se indicated into individual nanoparticle all for its own as well as the bimetallic nanoparticle chart presented all elements. With the other words, the bimetallic EDX chart contains the sum of all the above-mentioned atoms. Obviously, these results affirmed the composition of bimetallic and agreed with all the above characterizations, which included physiochemical and morphological analysis as well as a clear description of a green process used in the biosynthesis of NPs and confirmation of the integration that takes place. In this context, the elemental percentages were presented in Fig. 4f that observed the elemental constituent percentage of individual and bimetallic NPs that affirmed biosyntheis of individual and bimetallic NPs.

### 3.3. Biological activities

#### 3.3.1. Antibacterial activity

**Fig. 5:**
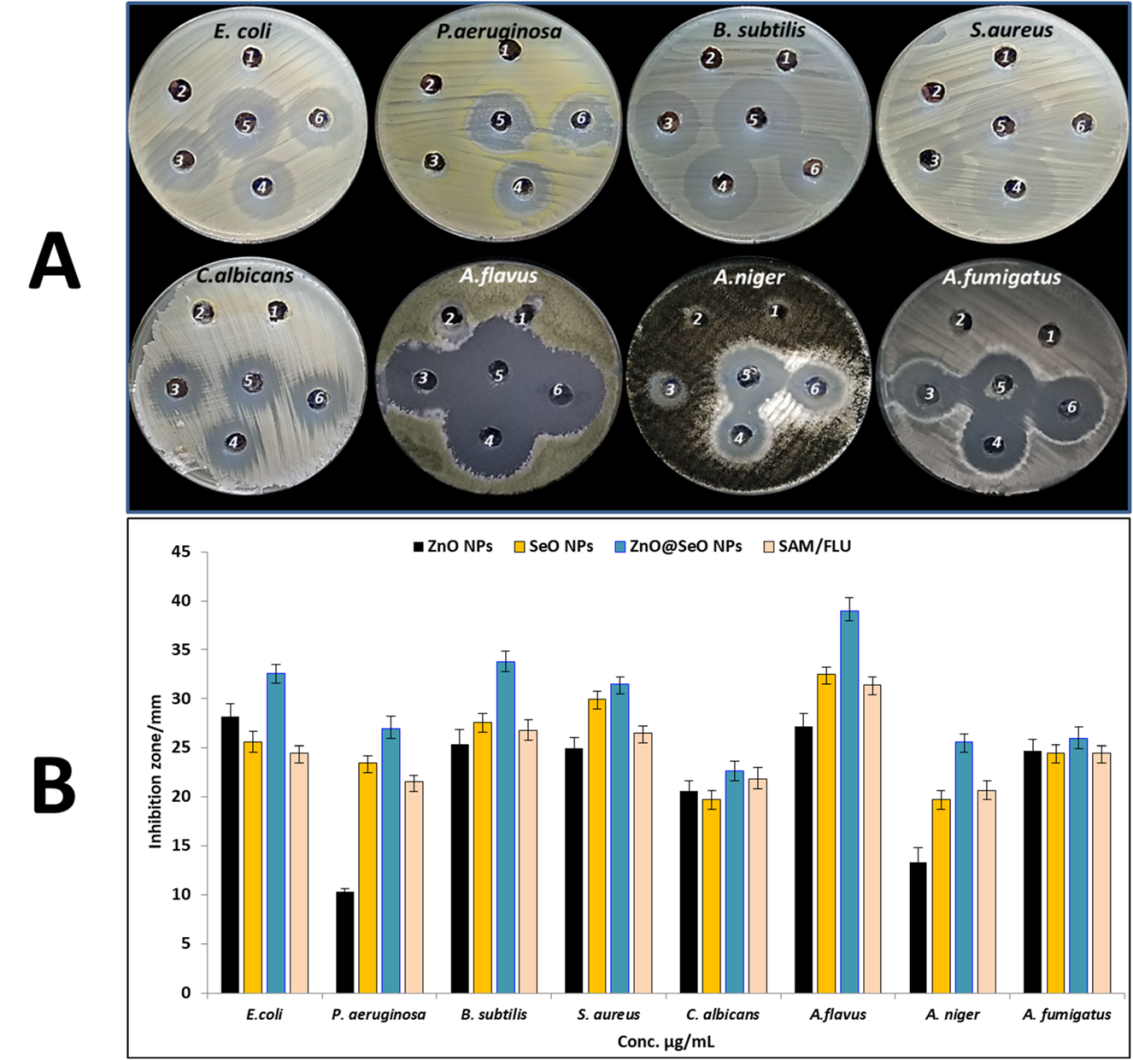
Antimicrobial activity of zinc acetate (1), Selenium oxide (2), ZnO NPs (3), SeO NPs (4), ZnO@SeO NPs (5) and AMC/FLU (6) using agar well diffusion method (A), and inhibition zone (B).

The use of highly potent antimicrobial medications is required to regain control of the invasion of novel bacterial infections, the increasing proliferative capacities of these infections, and antibacterial resistance, all of which have serious consequences for public health. Bimetallic NPs, created by mixing two different metals, have lately surfaced as having a promising antimicrobial efficiency that outperforms monometallic counterparts. This is because bimetallic NPs have synergistic effects, a wide range of physiochemical properties, and a variety of modes of action. Consequently, bimetallic ZnO@SeO NPs were synthesized in the current study and evaluated for antibacterial as well as antifungal activity. Antibacterial activity of bimetallic ZnO@SeO NPs, ZnO NPs, SeO NPs, and other start precursors was assessed against tested bacterial, as shown in Fig. 5 and Table 1. Results revealed that all prepared metal NPs in this study exhibited antibacterial activity against Gram-negative and Gram-positive bacteria (Fig. 5), where the antibacterial activity of bimetallic ZnO@SeO NPs was higher than that of both ZnO NPs and SeO NPs. Moreover, biosynthesized ZnO NPs and SeO NPs exhibited moderate antibacterial activity against *E. coli*, *P. aeruginosa*, *B. subtilis* and *S. aureus* where inhibition zones (28.2, 9.5, 25.3 and 25 mm) and (25.6, 23.5, 27.6 and 29.9 mm) respectively. Furthermore, bimetallic ZnO@SeO NPs exhibited promising antibacterial activity where was higher than ZnO NPs and SeO NPs, where inhibition zones were 32.6, 26.9, 33.8 and 31.5 mm respectively. Likewise, MIC results for bimetallic ZnO@SeO NPs was better than ZnO NPs and SeO NPs, where MICs of bimetallic ZnO@SeO NPs toward *E. coli*, *P. aeruginosa*, *B. subtilis* and *S. aureus* were 3.9, 15.62, 3.9 7.81 μg ml^−1^. Also, MICs of ZnO NPs, SeO NPs were (15.62, 1000, 32.25 & 31.25 μg ml^−1^) and (62.5, 62.5, 31.25 & 31.25 μg ml^−1^) respectively. On the other hand, starting precursors (zinc acetate and selenium oxide) and PPE did not exhibit any activity on all tested bacterial strains.

**Table 1:** Inhibition zones and MICs of bimetallic ZnO@SeO NPs, ZnO NPs, SeO NPs and other start precursors

Test microorganism	Zn^+^	ZnO NPs		Se^+^	SeO NPs		ZnO@SeO NPs		AMC/FLU
	IZ (mm)	IZ (mm)	MIC μg ml^−1^	IZ (mm)	IZ (mm)	MIC μg ml^−1^	IZ (mm)	MIC μg ml^−1^	IZ (mm)
*E. coli*	ND	28.2 ± 1.26	15.62	ND	25.6 ± 1.10	62.5	32.6 ± 0.85	3.9	24.5 ± 0.71
*P. aeruginosa*	ND	9.5 ± 0.68	1000	ND	23.5 ± 0.71	62.5	26.9 ± 1.27	15.62	21.6 ± 0.57
*B. subtilis*	ND	25.3 ± 1.53	31.25	ND	27.6 ± 0.85	31.25	33.8 ± 1.06	3.9	26.75 ± 1.06
*S. aureus*	ND	25.0 ± 1.00	31.25	ND	29.9 ± 0.90	31.25	31.5 ± 0.71	7.81	26.5 ± 0.71
*C. albicans*	ND	20.6 ± 1.04	62.5	ND	19.7 ± 0.99	125	22.7 ± 0.92	31.25	21.85 ± 1.20
*A. flavus*	ND	27.2 ± 1.31	15.62	ND	32.5 ± 0.71	15.62	39.0 ± 1.34	1.95	31.4 ± 0.85
*A. niger*	ND	13.3 ± 1.53	250	ND	19.7 ± 0.92	125	25.6 ± 0.78	15.62	20.7 ± 0.99
*A. fumigatus*	ND	24.7 ± 1.15	31.25	ND	24.5 ± 0.78	62.5	25.9 ± 1.20	15.62	24.5 ± 0.71

Bimetallic NPs composed of two different metals can exhibit synergistic effects, where the combined action of the metals enhances the antibacterial activity. The interaction between the metals can result in increased ROS generation, improved metal ion release, and enhanced physical damage to bacterial cells.^58^ Bimetallic NPs can physically damage bacterial cells; the small size and high surface area of NPs allow them to interact with the bacterial cell membrane, also interaction between positively charged NPs and negatively charged bacterial cell membranes leading to its disruption and leakage of intracellular components, then inhibition of bacterial growth and cell death.^59–61^ Also, metallic ions cause disturbances in hemostasis where they bind to the peptidoglycan layer's SH groups, causing the cell wall to break down.^62^ Moreover, metallic NPs produce ROS which can cause oxidative stress in bacterial cells by damaging proteins, lipids, and DNA. The accumulation of ROS can disrupt cellular processes and lead to bacterial cell death. Aggregate on bacterial cell membranes and produce reactive oxygen species (ROS), which cause cell death.^63^

#### 3.3.2. Antifungal activity

Antifungal activity of bimetallic ZnO@SeO NPs, ZnO NPs, SeO NPs and other start precursors was assessed against tested fungal strains using agar well diffusion method as shown in Fig. 5 and Table 1. Results illustrated that, bimetallic ZnO@SeO NPs appeared the highest antifungal activity among other prepared NPs. Inhibition zones of bimetallic ZnO@SeO NPs at concentration 1000 μg ml^−1^ against *C. albicans*, *A. flavus*, *A. fumigatus* and *A. niger* were 22.7, 39.0, 25.9 and 25.6 mm respectively. Moreover, *A. flavus* was the most sensitive fungus among other tested fungal strains where MIC was 1.95 μg ml^−1^, while as MIC toward others was in range 15.62–31.25 μg ml^−1^. Furthermore, both ZnO NPs, SeO NPs revealed antifungal activity against all tested strains but was lower than bimetallic ZnO@SeO NPs. Inhibition zones of ZnO NPs, SeO NPs at concentration 1000 μg ml^−1^ against *C. albicans*, *A. flavus*, *A. fumigatus* and *A. niger* were (20.6, 27.2, 24.7 & 13.3 mm) and (19.7, 32.5, 24.5 & 19.7 mm) respectively. Also, MICs of ZnO NPs, SeO NPs were (62.5, 15.62, 31.25 & 250 μg ml^−1^) and (125, 15.62, 62.5 & 125 μg ml^−1^) respectively. On the other hand, precursors (zinc acetate and selenium oxide) and PPE did not exhibit any activity on all tested fungal strains, this confirms the activity of bimetallic ZnO@SeO NPs, ZnO NPs, SeO NPs attributed only for metals in nanoform. All types of fungi have cell walls and membranes; the walls of the cells contain mannoproteins, glucan-chitin, glucan, and mannoprotein. Bimetallic NPs have the potential to exert fungicidal as well as fungistatic effects. The inhibition of beta-glucan synthase and, as a consequence, the cell wall is what ultimately leads to the fungistatic effect. While the fungicidal effect is caused by changes in the wall integrity, which results in the loss of the wall's mechanical resistance and causes cell death as a result of differences in osmotic pressure, the osmotic pressure differences also play a role.^64^ Also bimetallic NPs can disrupt fungal cell division and growth, inhibit fungal enzyme activity, and interfere with DNA replication and repair processes. These disruptions can impair fungal cell viability and inhibit fungal growth.^60^

#### 3.3.3. Cytotoxicity and anticancer activity

**Fig. 6:**
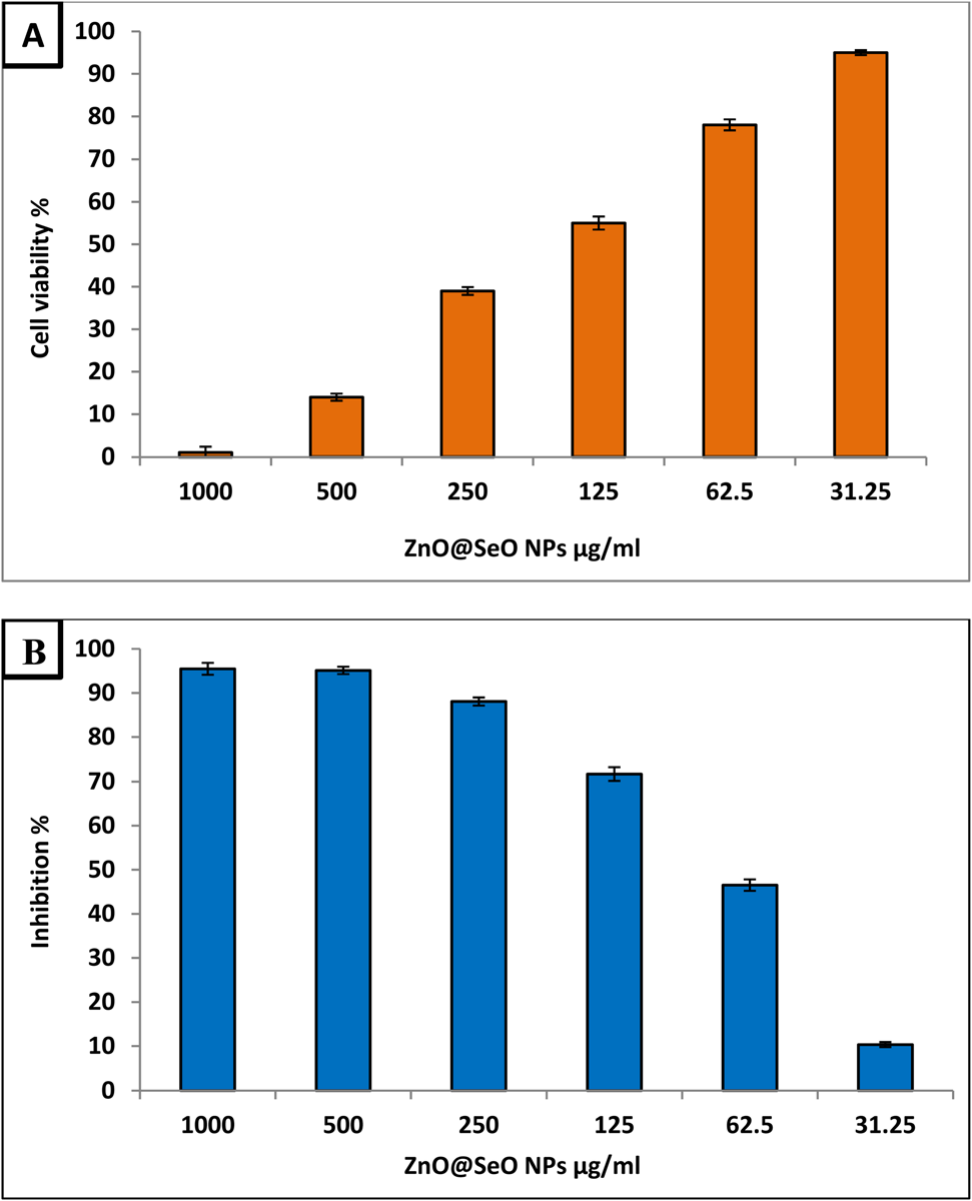
Cytotoxicity of Bimetallic ZnO@SeO NPs against Vero normal cell line (A) and Hep-G2 (B).

The initial step in determining the safety of these chemicals is to test their cytotoxicity against normal cell lines 50. As shown in Fig. 6A, the current work evaluated the cytotoxicity of ZnO@SeO NPs against the vero normal cell line. Results showed that ZnO@SeO NPs' IC50 value was 164 μg ml^−1^. In general, a substance is considered non-cytotoxic if the IC50 is less than 90 μg ml^−1^.^65^ Therefore, this compound is non-toxic and safe in use.

Bimetallic NPs have shown promise in the field of cancer research due to their potential anticancer activity.^66^ Therefore, the biosynthesized ZnO@SeO NPs in the current study were evaluated as anticancer agent toward Hep-G2 cancerous cell line at different concentration within range 1000–31.25 μg ml^−1^ as illustrated in Fig. 6B. Results revealed that the biosynthesized ZnO@SeO NPs exhibited promising anticancer activity toward Hep-G2 cell line where IC50 was 71.1 μg ml^−1^. Moreover, results illustrated that concentrations 125, 62.5 and 31.25 showed anticancer activity where inhibition percentages were 71.7, 46.5 and 10.3 μg ml^−1^. This confirms that biosynthesized ZnO@SeO NPs low concentrations can be used for combating cancers. The formation of reactive oxygen species (ROS) as a result of oxidative stress on cancer cells is hypothesized to be the mechanism of action of metal polymer nanocomposite as an anticancer agent. ROS can induce oxidative stress and cause damage to cancer cells by targeting cellular components like proteins, lipids, and DNA (DNA strand breaks, base modifications, and DNA cross-linking). The accumulation of ROS can lead to cell death through apoptosis or necrosis.^67^ In addition to this, it is possible that it is caused by the electrochemical interaction of certain negative charges on the surface of cancer cells with the positive charges of metal NPs in the environment.^68^ Furthermore, bimetallic NPs can disrupt the mitochondrial membrane potential, impair ATP production, and induce mitochondrial-mediated apoptosis, triggering cancer cell death.^69^ Moreover, NPs can interfere with signaling pathways involved in angiogenesis, such as VEGF (vascular endothelial growth factor), thereby preventing the blood supply to tumors and inhibiting their growth.^70^

## 4. Conclusion

In the current study, bimetallic ZnO@SeO NPs were successfully biosynthesized using PPE for the first time. The physicochemical analysis was declared for the biosynthesis of ZnO@SeO NPs and the topographical analysis in comparison with individual NPs with nanosizes of about 20, 30, and 40 nm for ZnO NPs, SeO NPs, and ZnO@SeO NPs, respectively. Furthermore, bimetallic ZnO@SeO NPs showed promising antibacterial activity against Gram-negative and Gram-positive bacteria. Moreover, it exhibited antifungal activity toward both unicellular and multicellular fungi. Moreover, it showed promising anticancer activity against Hep-G2 at safe concentrations.

## Data availability

The data and materials are available as per request.

## Author contributions

Amr H. Hashem: conceptualization, methodology, writing – original draft preparation, writing – review and editing; Abdulaziz A. Al-Askar: methodology, writing – original draft preparation; Mohammad Reza Saeb, Kamel A. Abd-Elsalam: writing – review and editing, critically revising; Ahmad S. El-Hawary: methodology, writing – original draft preparation, writing – review and editing; Mohamed S. Hasanin: conceptualization, methodology, writing – original draft preparation, writing – review and editing.

## Conflicts of interest

The authors declare that they have no conflict to interests.

## Supplementary Material
